# Classification of Benign and Malignant Breast Tumors in Ultrasound Images with Posterior Acoustic Shadowing Using Half-Contour Features

**DOI:** 10.1007/s40846-015-0031-x

**Published:** 2015-04-11

**Authors:** Zhuhuang Zhou, Shuicai Wu, King-Jen Chang, Wei-Ren Chen, Yung-Sheng Chen, Wen-Hung Kuo, Chung-Chih Lin, Po-Hsiang Tsui

**Affiliations:** 1 Biomedical Engineering Center, College of Life Science and Bioengineering, Beijing University of Technology, Beijing, 100124 China; 2 Department of Surgery, Cheng Ching General Hospital, Chung Kang Branch, Taichung, 407 Taiwan; 3 Department of Surgery, National Taiwan University Hospital, Taipei, 10048 Taiwan; 4 Department of Electrical Engineering, Yuan Ze University, Chung Li, 32003 Taiwan; 5 Department of Computer Science and Information Engineering, Chang Gung University, Taoyuan, 33302 Taiwan; 6 Department of Medical Imaging and Radiological Sciences, College of Medicine, Chang Gung University, Taoyuan, 33302 Taiwan; 7 Institute of Radiological Research, Chang Gung University and Hospital, Taoyuan, 33302 Taiwan

**Keywords:** Ultrasound, Breast tumor, Posterior acoustic shadowing (PAS), Half-contour feature, Standard deviation of degree (SDD)

## Abstract

Posterior acoustic shadowing (PAS) can bias breast tumor segmentation and classification in ultrasound images. In this paper, half-contour features are proposed to classify benign and malignant breast tumors with PAS, considering the fact that the upper half of the tumor contour is less affected by PAS. Adaptive thresholding and disk expansion are employed to detect tumor contours. Based on the detected full contour, the upper half contour is extracted. For breast tumor classification, six quantitative feature parameters are analyzed for both full contours and half contours, including standard deviation of degree (SDD), which is proposed to describe tumor irregularity. Fifty clinical cases (40 with PAS and 10 without PAS) were used. Tumor circularity (TC) and SDD were both effective full- and half-contour parameters in classifying images without PAS. Half-contour TC [74 % accuracy, 72 % sensitivity, 76 % specificity, 0.78 area under the receiver operating characteristic curve (AUC), *p* > 0.05] significantly improved the classification of breast tumors with PAS compared to that with full-contour TC (54 % accuracy, 56 % sensitivity, 52 % specificity, 0.52 AUC, *p* > 0.05). Half-contour SDD (72 % accuracy, 76 % sensitivity, 68 % specificity, 0.81 AUC, *p* < 0.05) improved the classification of breast tumors with PAS compared to that with full-contour SDD (62 % accuracy, 80 % sensitivity, 44 % specificity, 0.61 AUC, *p* > 0.05). The proposed half-contour TC and SDD may be useful in classifying benign and malignant breast tumors in ultrasound images affected by PAS.

## Introduction

Breast cancer is a health problem for women worldwide [1]. Ultrasound has become a useful adjunct modality for breast tumor diagnosis because it is cost-effective, noninvasive, and performed in real time [2]. However, certain inherent characteristics of ultrasound images, including low contrast, speckle noise, and tissue-related textures, may cause difficulties for radiologists [3]. Moreover, inter- or intraobserver differences in the understanding and diagnosis of ultrasound images might occur [4]. Hence, developing a computer-aided detection/diagnosis (CAD) system for analyzing ultrasound images is crucial [3, 5–7]. The diagnosis result of a CAD system can provide a second opinion for radiologists in the detection and diagnosis of breast lesions [4, 8, 9].

Typically, an ultrasound CAD system is composed of four parts: preprocessing, segmentation, feature extraction and selection, and classification [10]. Breast ultrasound image segmentation techniques include histogram thresholding [11], region growing [11, 12], model-based methods (such as active contour models or snakes [12, 13], level sets [14], and Markov random fields [15]), graph-based methods [16–18], neural networks [11, 19, 20], and watersheds [21, 22]. For ultrasound image classification, many features have been proposed to describe breast tumors, such as shape, margin, calcification, echogenicity, posterior echo, and echo texture. The features of ultrasound images can be classified into four categories: texture features, morphologic features, model-based features, and descriptor features [10]. Identifying most of these features relies on accurate segmentation of tumor contours, but ultrasound image segmentation and classification can be difficult when posterior acoustic shadowing (PAS) occurs. PAS occurs in ultrasound images when a strong attenuation effect caused by the tumor growth weakens the strength of the ultrasonic beam. PAS is frequently observed for malignant tumors [5, 23], and can bias the computerized segmentation and classification of breast tumors in ultrasound images. However, few of the current ultrasound image segmentation or classification techniques address the PAS problem [3, 5–7, 10–23].

Since the upper half of the tumor contour is less affected by the PAS effect than is the lower half, the present study proposes using features based on the upper half contour to classify benign and malignant breast tumors in ultrasound images with PAS. Adaptive thresholding and disk expansion (DE) [24] are employed for breast tumor segmentation, and half-contour feature parameters are analyzed for breast tumor classification. To evaluate the effectiveness of the proposed method, 50 clinical cases were obtained from a hospital to calculate half-contour feature parameters. A receiver operating characteristic (ROC) curve was plotted to evaluate the method, and a *t* test was applied to estimate the diagnostic performance of this method.

## Materials and Methods

### Data Acquisition

This study was approved by the Institutional Review Board of National Taiwan University Hospital and the patients signed informed consent forms. Ultrasound images were collected using a commercial portable ultrasound scanner (Model 3000, Terason, Burlington, MA, USA). The probe comprised a wideband linear array with a central frequency of 7.5 MHz and 256 elements. Fifty female patients volunteered to participate in the study. A sonographer performed the ultrasound scanning, and breast tumors were identified as benign (fibroadenoma) or malignant (invasive ductal carcinoma) according to biopsy reports. There were 40 cases with PAS and 10 cases without PAS.

### Breast Tumor Segmentation

Let *I* denote an ultrasound image with *M* × *N* pixels and *I*(*x*, *y*) denote a pixel at (*x*, *y*). The threshold *TH*_*x*_ for each column of *I* is defined as:

1 $$TH_{x} = \frac{1}{N}\sum\limits_{y = 1}^{N} {I(x,\;y)} .$$

The image *I* can then be binarized with *TH*_*x*_ to form a binary image *B*:

2 $$B(x,\;y) = \left\{ {\begin{array}{ll} {0}, &\quad {{\text{if}}\;I(x,\;y) < TH_{x} }, \\ {1}, &\quad {{\text{otherwise}}}. \\ \end{array} } \right.$$

To incorporate regional information into the binarization, the mean gray value $\bar{I}(x,\;y)$ in a neighboring window of size (2*n* + 1) × (2*n* + 1), *n* = 1, 2, 3,…, is considered:

3 $$\bar{I}(x,\;y) = \frac{1}{{(2n + 1)^{2} }}\sum\limits_{i = - n}^{n} {\sum\limits_{j = - n}^{n} {I(x + i,\;j + y)}}.$$

Adaptive thresholding is performed to obtain the binary image *B*:

4 $$B(x,\;y) = \left\{ {\begin{array}{ll} {0}, &\quad {{\text{if}}\;I(x,\;y) < TH_{x} \quad {\text{and}}\quad \bar{I}(x,\;y) < TH_{x} }, \\ {1}, &\quad {{\text{otherwise}}}. \\ \end{array} } \right.$$

**Fig. 1:**
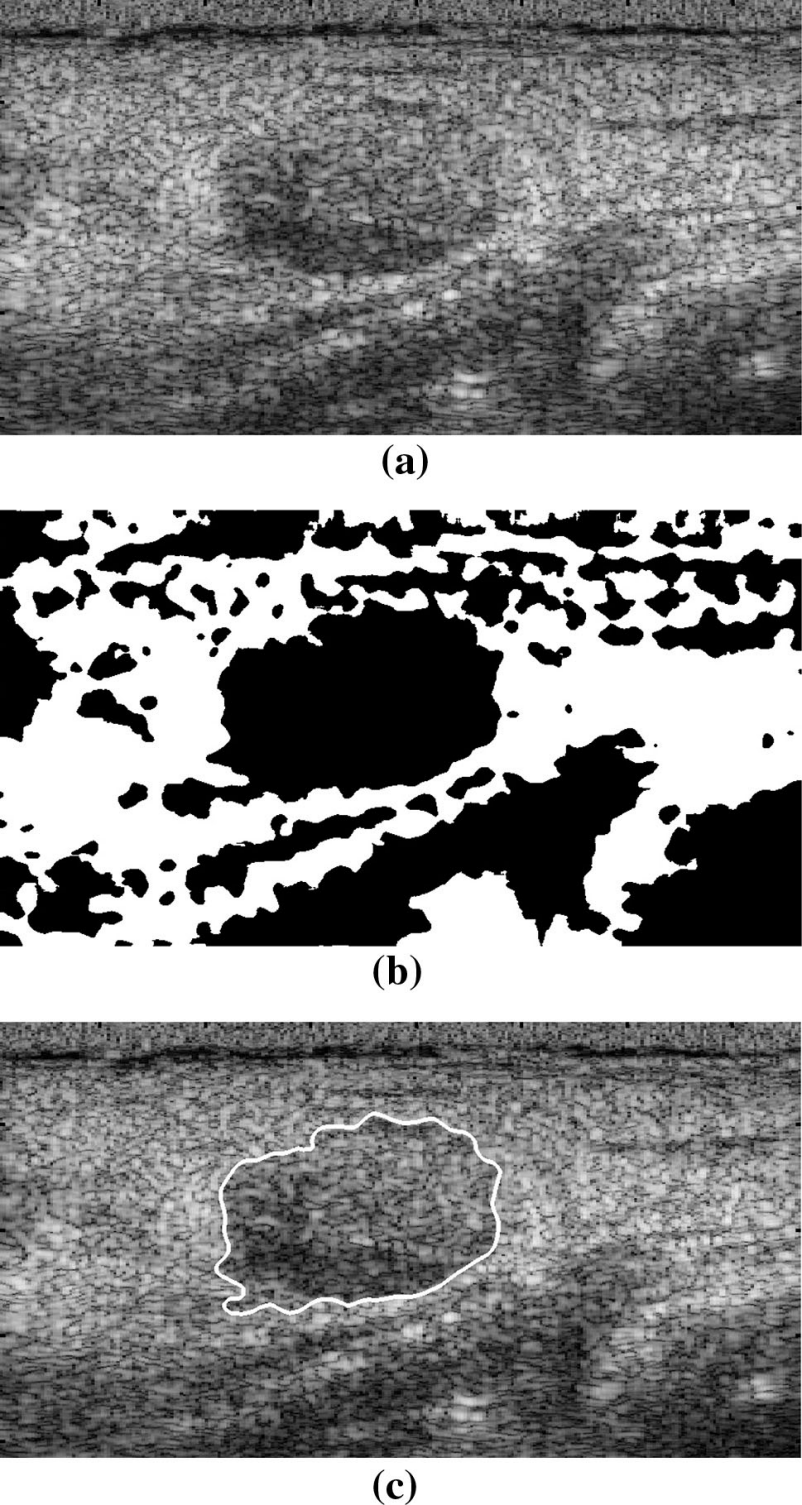
**a** Breast ultrasound image. **b** Image after adaptive thresholding. **c** Tumor contour extracted using DE

The DE segmentation method [24] is employed to detect the tumor contour in the binary image *B*. Figure 1b shows the result of using the proposed adaptive thresholding for an ultrasound image. Figure 1c shows the tumor contour extracted from the ultrasound image using DE.

### Breast Tumor Classification

**Fig. 2:**
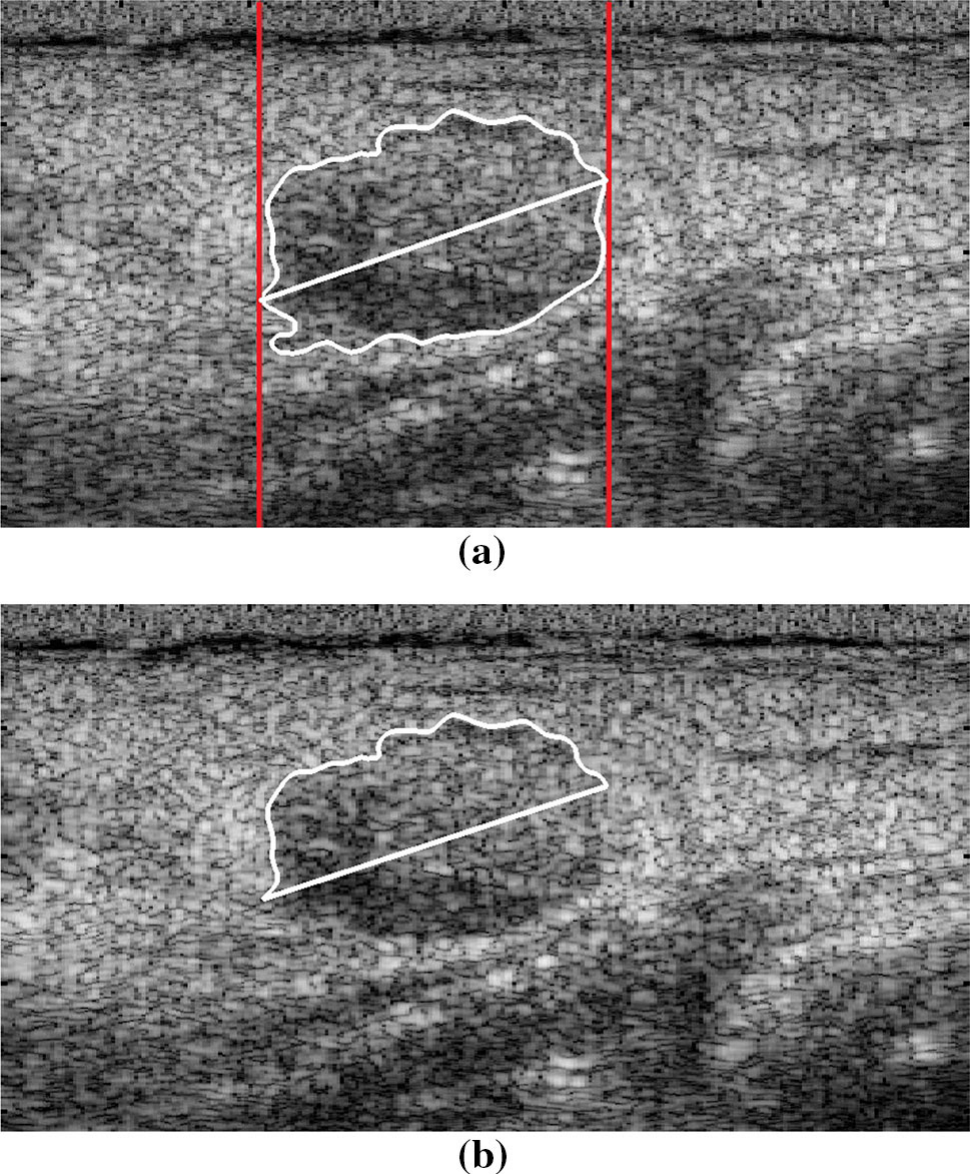
**a** Leftmost and rightmost pixels (indicated by the *two red lines*) detected from the extracted tumor full contour. **b** Detected tumor half contour

In the ultrasound image with the extracted tumor contour *C*, the half contour was detected by determining the leftmost and rightmost pixels *P*_*l*_ and *P*_*r*_, respectively, of the tumor region, excluding the portion affected by PAS, as shown in Fig. 2. A line *L*_*lr*_ was drawn to connect *P*_*l*_ and *P*_*r*_. The half contour was defined as the upper part of *C*, between *P*_*l*_ and *P*_*r*_.

The features of the tumor half contour, including its shape and margin, are described using six typical feature parameters: tumor circularity (TC), mean of the normalized radial length (NRL_M_), standard deviation of the NRL (NRL_STD_), area ratio (AR), roughness index (RI), and standard deviation of degree (SDD). TC, NRL_M_, NRL_STD_, and AR are parameters that describe tumor shape (the degree of irregularity), RI describes the margin (the degree of spiculation), and SDD is a contour feature parameter proposed here to characterize the half-contour irregularity. To compare the six half-contour features with full-contour features, six full-contour feature parameters were also calculated.

TC is a gross contour feature descriptor that has been shown to be useful in classifying breast masses [25]. TC was calculated using:

5 $$TC = \frac{{P^{2} }}{A},$$

where *P* is the perimeter and *A* is the area of the tumor half contour. The perimeter *P* was measured by summing the number of pixels corresponding to the tumor contour, and the area *A* was calculated by the number of pixels inside the contour.

Similar to TC, NRL_M_, and NRL_STD_ reflect macroscopic boundary changes, but they can also indicate subtle boundary changes [26]. NRL was first computed using:

6 $$\hat{d}(i) = \frac{d(i)}{{\max} (d)},$$

where *d*(*i*) denotes the distance from the *i*th contour pixel to the tumor centroid. NRL_M_ and NRL_STD_ were then respectively computed using:

7 $$\bar{d} = \frac{1}{N}\sum\limits_{i = 1}^{N} {\hat{d}(i)} ,$$

and

8 $$\sigma_{d} = \sqrt {\frac{1}{N - 1}\sum\limits_{i = 1}^{N} {\left( {\hat{d}(i) - \bar{d}} \right)^{2} } } ,$$

where *N* is the number of contour pixels.

AR is a measure of the percentage of the tumor located outside the circular region defined by the mean of the *x*–*y* line plot [26]. It was computed using:

9 $$A = \frac{1}{{\bar{d}N}}\sum\limits_{i = 1}^{N} {\left( {\hat{d}(i) - \bar{d}} \right)} ,$$

where $$\hat{d}(i) - \bar{d} = 0,$$ $$\forall \hat{d}(i) \le \bar{d}.$$

RI represents the degree of tumor spiculation and was computed using [26]:

10 $$R = \frac{1}{N}\sum\limits_{i = 1}^{N} {|\hat{d}(i) - \hat{d}(i + 1)|} .$$

To clearly describe the shape irregularity of the tumor half contour, SDD is proposed to classify tumors. *θ*_*s*_ is defined as the relationship between the *s*th contour pixel and the (*s* *−* *k*)th and (*s* + *k*)th pixels:

11 $$\theta_{s} = \cos^{ - 1} \left( {\frac{{\overrightarrow {{M_{s} M_{s - k} }} \cdot \overrightarrow {{M_{s} M_{s + k} }} }}{{|\overrightarrow {{M_{s} M_{s - k} }}| |\overrightarrow {{M_{s} M_{s + k} }} |}}} \right),$$

where $\overrightarrow {{M_{s} M_{s - k} }}$ denotes the position vector from the *s*th to the (*s* *−* *k*)th pixels, and $|\overrightarrow {{M_{s} M_{s - k} }} |$ is the length of the vector. SDD is defined as the standard deviation of *θ*_*s*_:

12 $$\sigma_{s} = \sqrt {\frac{1}{N}\sum\limits_{s = 1}^{N} {\left( {\theta_{s} - \bar{\theta }} \right)^{2} } } ,$$

where $\bar{\theta }$ is the mean of *θ*_*s*_.

### Statistical Analysis

By using *t*-tests, the probability value (*p* value) of each of the six feature parameters was determined to classify benign and malignant breast tumors. The accuracy, sensitivity, and specificity of the features were then respectively computed using:

13 $$\begin{aligned} Accuracy &= \frac{TP + TN}{TP + TN + FP + FN}, \\ Sensitivity &= \frac{TP}{TP + FN}, \\ Specificity &= \frac{TN}{TN + FP}, \\ \end{aligned}$$

where *TP* (true positive) denotes the number of malignant cases truly classified as positive, *TN* (true negative) denotes the number of benign cases truly classified as negative, *FP* (false positive) denotes the benign cases falsely classified as positive, and *FN* (false negative) denotes the malignant cases falsely classified as negative. The tumor classification performance of each feature parameter was evaluated using the ROC curve.

## Results

### Classification of Breast Tumors Without PAS

**Fig. 3:**
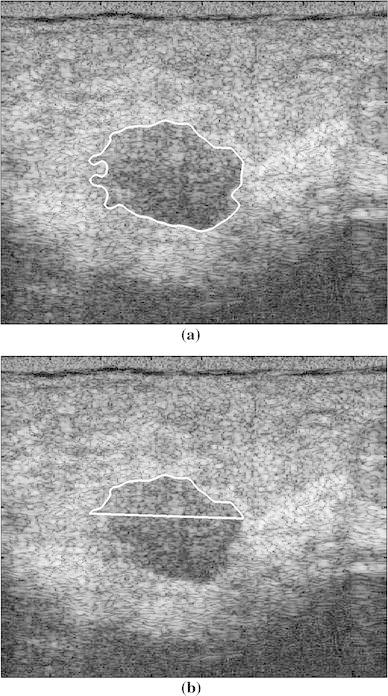
Benign case without shadowing. **a** Full contour and **b** half contour

**Fig. 4:**
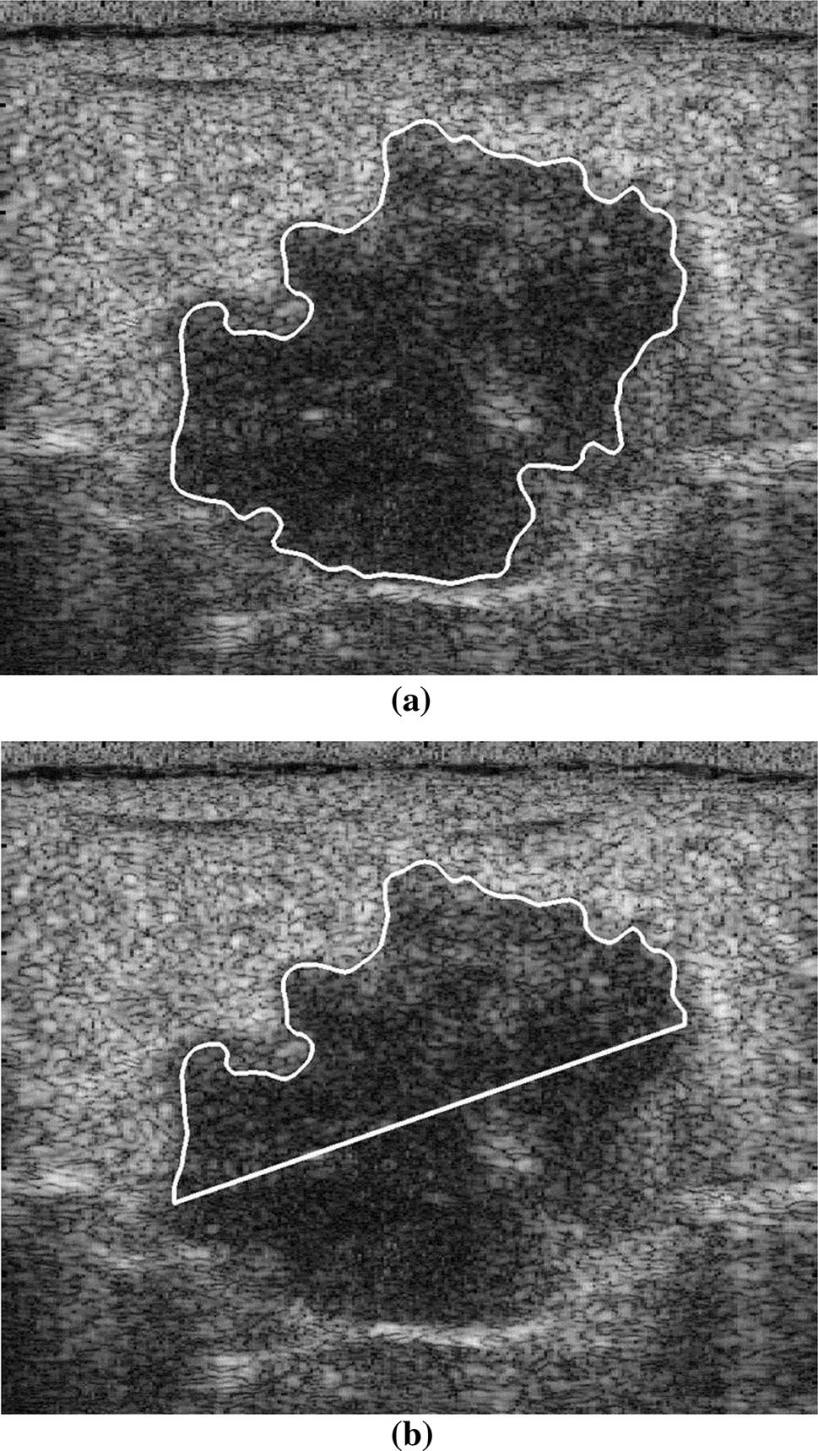
Malignant case without shadowing. **a** Full contour and **b** half contour

The feasibility of using the six feature parameters in discriminating between benign and malignant breast tumors was validated using images without PAS. The full- and half-contour features were identified for the 10 cases without PAS. One benign case and one malignant case without PAS are shown in Figs. 3 and 4, respectively. The accuracy, sensitivity, and specificity of each feature parameter in classifying benign and malignant breast tumors was analyzed using images without PAS (*n* = 10). The results are shown in Table 1. Among the six feature parameters, TC and SDD were most effective for classifying tumors without PAS, both for full contours and half contours.

**Table 1:** Accuracy, sensitivity, and specificity of each parameter in classifying benign and malignant breast tumors without PAS (*n* = 10)

Parameters	Accuracy (%)	Sensitivity (%)	Specificity (%)
*TC*			
Full contour	90	100	80
Half contour	100	100	100
*σ*_*s*_			
Full contour	90	100	80
Half contour	90	80	100
$$\bar{d}$$			
Full contour	60	40	80
Half contour	60	80	40
*σ*_*d*_			
Full contour	60	80	40
Half contour	50	20	80
*A*			
Full contour	60	80	40
Half contour	60	100	20
*RI*			
Full contour	80	80	80
Half contour	80	60	100

*TC* tumor circularity, *σ*_*s*_ standard deviation of degree, $\bar{d}$ mean of the normalized radial length, *σ*_*d*_ standard deviation of the normalized radial length, *A* area ratio, *RI* roughness index

### Classification of Breast Tumors with PAS

**Fig. 5:**
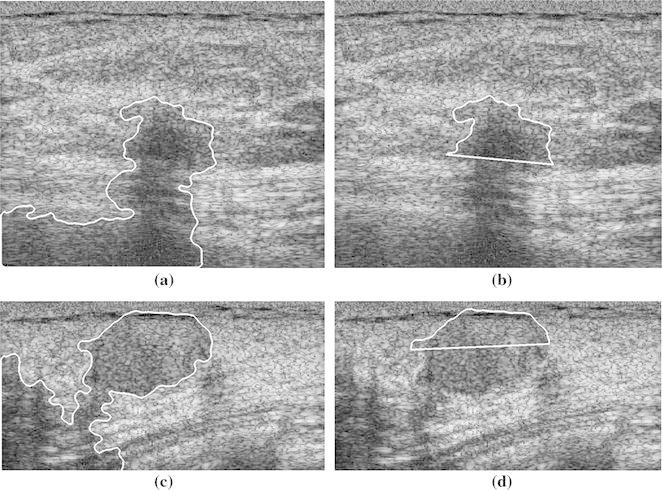
Two benign cases with shadowing. **a**, **c** Full contour and **b**, **d** half contour

**Fig. 6:**
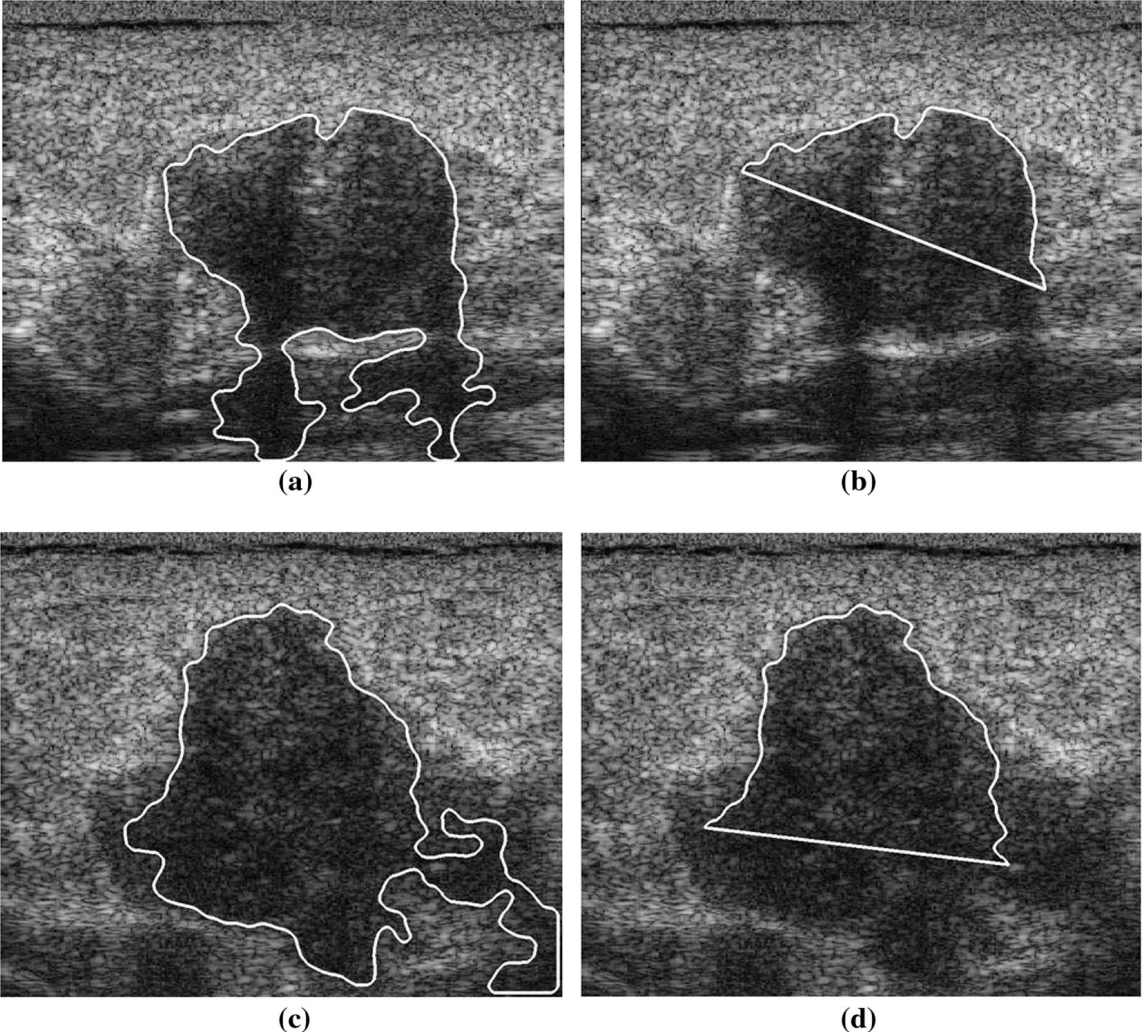
Two malignant cases with shadowing. **a**, **c** Full contour and **b**, **d** half contour

As TC and SDD yielded the best performance for breast tumors without PAS, these two parameters were evaluated in discriminating between benign and malignant tumors in images with PAS. Two benign cases and two malignant cases with PAS are shown in Figs. 5 and 6, respectively. Table 2 shows the accuracy, sensitivity, and specificity of the two parameters (*n* = 50). Half-contour TC was significantly more accurate, sensitive, and specific than full-contour TC, indicating the capability of using half-contour TC to manage the PAS effect. Half-contour SDD was slightly less sensitive, but considerably more accurate and specific than full-contour SDD. This reflects the capability of using the half-contour SDD to discriminate between benign and malignant breast tumors in images with PAS.

**Table 2:** Accuracy, sensitivity, and specificity of TC and SDD in classifying benign and malignant breast tumors with PAS (*n* = 50)

Performance parameters	Accuracy (%)	Sensitivity (%)	Specificity (%)
*TC*			
Full contour	54	56	52
Half contour	74	72	76
*σ*_*s*_			
Full contour	62	80	44
Half contour	72	76	68

*TC* tumor circularity, *σ*_*s*_ standard deviation of degree

**Fig. 7:**
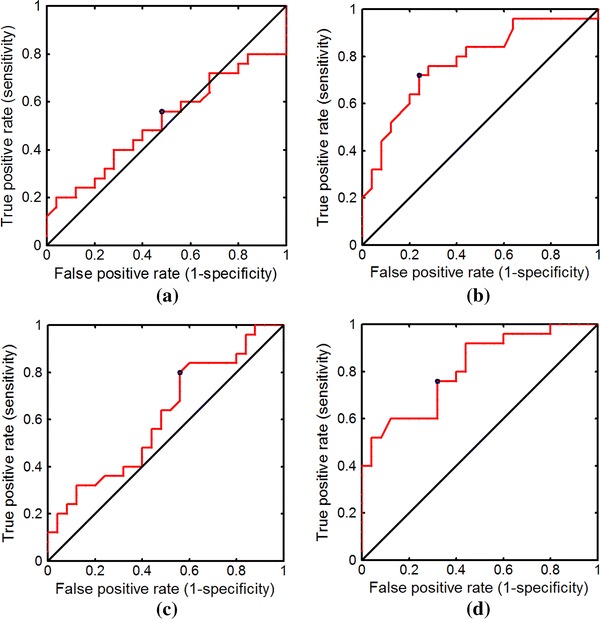
ROC curves obtained using **a** full- and **b** half-contour TC and **c** full- and **d** half-contour SDD to classify benign and malignant tumors with PAS

**Fig. 8:**
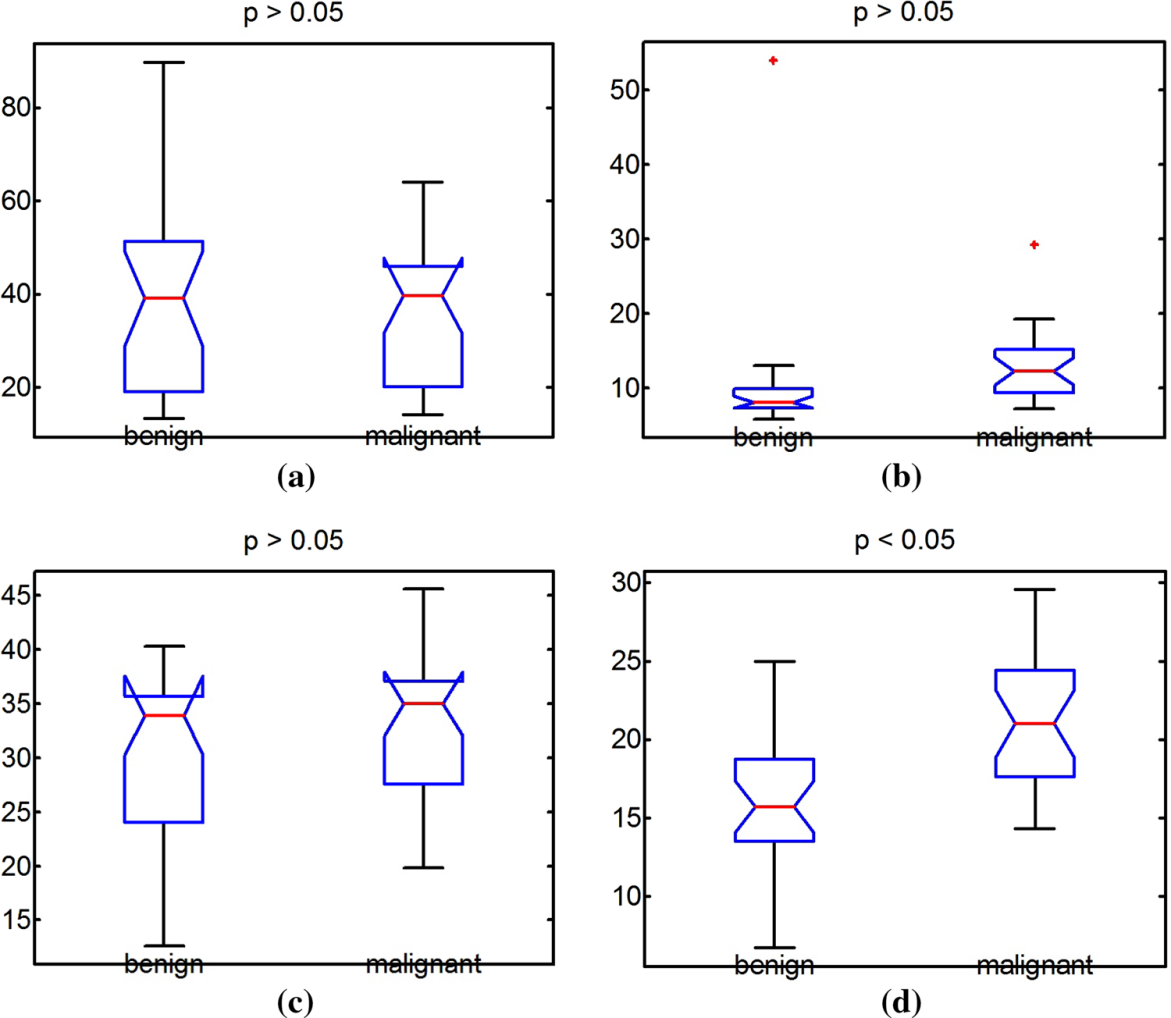
Results of *t*-test obtained using **a** full- and **b** half-contour TC and **c** full- and **d** half-contour SDD to classify benign and malignant tumors with PAS

Figure 7a shows the ROC curve for full-contour TC. Full-contour TC presented a low discrimination accuracy because it was affected by the PAS effect; its area under the ROC curve (AUC) was only 0.52. Figure 7b shows that half-contour TC can be used for classifying breast tumors more accurately, with an AUC of 0.78. Figure 7c, d show the ROC curves of the half- and full-contour SDD, respectively; half-contour SDD presented a higher AUC (0.81) than that of full-contour SDD (0.61). As the ROC curves show, half-contour TC and half-contour SDD may be effectively used to classify benign and malignant breast tumors. Figure 8 shows the *t*-test results of using the full- and half-contour TCs as well as full- and half-contour SDDs on images with PAS. Half-contour SDD had a *p* value of <0.05, indicating discrimination capability, whereas full-contour TC and SDD both had *p* values of >0.05.

## Discussion

As ultrasonic waves propagate through biological tissue, their energy is reduced or attenuated exponentially with depth. The energy is lost because of absorption, scattering, and specular reflection at the boundary between two layers of tissue. When little energy is transmitted through a mass, such as a solid tumor composed of mainly connective tissues, an acoustic shadow may be created behind such a mass [27]. The energy attenuation coefficient increases with the ultrasonic transmission frequency. A higher frequency is required to obtain a higher ultrasonic imaging resolution. The PAS effect can affect ultrasound image segmentation, and thus may affect the tumor contour-based classification of benign and malignant breast tumors.

The present study proposes using half-contour feature parameters to classify benign and malignant breast tumors in ultrasound images with PAS. It was demonstrated that full-contour features are inadequate for managing PAS. Half-contour features improve the accuracy, sensitivity, and specificity in classifying benign and malignant breast tumors. Our findings indicate that PAS may be addressed by using half-contour features to diagnose breast tumors using ultrasound images. The half contour can be delineated manually by a radiologist, or partially or fully extracted automatically by a computer. However, to reduce inter- and intraoperator variance, fully automated breast tumor segmentation is preferable. Additional studies on the half-contour analysis of ultrasound images with PAS are necessary.

It should be noted that computerized breast tumor segmentation of ultrasound images remains a challenging task, due to such factors as the partial volume effect and the side acoustic shadow. The DE segmentation method used in this study could yield acceptable half contours according to the identification by the physician, and the half-contour features have been demonstrated to be capable of classifying benign and malignant breast tumors with PAS. The segmentation and classification accuracy of breast tumors may be increased by improving the DE method or employing more accurate segmentation methods. Nevertheless, this paper focuses on the feasibility of using half-contour features in differentiating begin tumors from malignant ones. In the future, more advanced breast tumor segmentation techniques can be studied to improve the recognition accuracy of breast tumors with PAS.

**Fig. 9:**
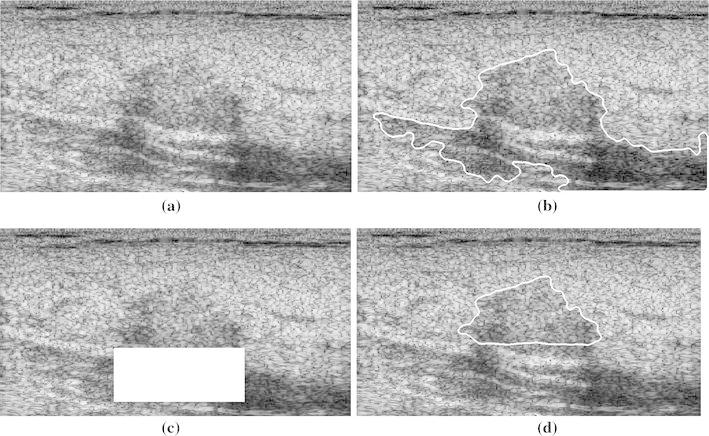
**a** Breast ultrasound image. **b** Tumor full contour extracted using adaptive thresholding and DE. **c** PAS part excluded to detect half contour. **d** Detected tumor half contour

One limitation of the proposed method is that the leftmost and rightmost pixels used to determine the half contour may lie in an area with PAS, resulting in biased half-contour detection, as shown in Fig. 9b. In certain cases, a manual exclusion operation may be necessary to detect the tumor half contour, as shown in Fig. 9c, d. This manual interaction may render half-contour detection operator-dependent. Another limitation of this study is the limited number of breast tumor cases and categories, and the large overlap between benign and malignant breast tumors. Additionally, the DE segmentation workflow was offline and time-consuming. To promote the proposed method toward clinical application, online processing is required. A potential approach is to use graphics processing unit-based parallel computing.

It is worthwhile to discuss different categories of breast tumors. There are a vast variety of malignant carcinomas, including invasive lobular carcinoma, medullary carcinoma, carcinoma in situ, mucinous carcinoma, and other tumors with abundant mucin. Benign tumors include adenosis, hyperplastic nodule, and adenomatous hyperplasia, whose incidence is high. Phyllodes tumors are also an important variety which can be benign, boarderline, or malignant. They are difficult to distinguish from fibroadenoma. Therefore, a higher number of inclusion cases and categories of disease should be considered in future studies.

## Conclusion

This paper proposed using half-contour features to classify benign and malignant breast tumors in ultrasound images with PAS. Two half-contour parameters, TC and SDD, enabled breast tumors to be classified more accurately in ultrasound images affected by PAS. In future studies, a higher number of tumor cases and categories should be considered, and the full automation of the tumor half-contour detection process, especially regarding images with PAS, should be explored.
